# Alzheimer’s disease in people with Down’s syndrome: the prospects for and the challenges of developing preventative treatments

**DOI:** 10.1007/s00415-016-8308-8

**Published:** 2016-10-24

**Authors:** Paula Castro, Shahid Zaman, Anthony Holland

**Affiliations:** Cambridge Intellectual and Developmental Disabilities Research Group, Department of Psychiatry, University of Cambridge, 18b Trumpington Road, Cambridge, CB2 8AH UK

**Keywords:** Down syndrome, Alzheimer’s disease, Treatment, Biomarkers

## Abstract

People with Down’s syndrome (DS) are at high risk for developing Alzheimer’s disease (AD) at a relatively young age. This increased risk is not observed in people with intellectual disabilities for reasons other than DS and for this reason it is unlikely to be due to non-specific effects of having a neurodevelopmental disorder but, instead, a direct consequence of the genetics of DS (trisomy 21). Given the location of the amyloid precursor protein (APP) gene on chromosome 21, the amyloid cascade hypothesis is the dominant theory accounting for this risk, with other genetic and environmental factors modifying the age of onset and the course of the disease. Several potential therapies targeting the amyloid pathway and aiming to modify the course of AD are currently being investigated, which may also be useful for treating AD in DS. However, given that the neuropathology associated with AD starts many years before dementia manifests, any preventative treatment must start well before the onset of symptoms. To enable trials of such interventions, plasma, CSF, brain, and retinal biomarkers are being studied as proxy early diagnostic and outcome measures for AD. In this systematic review, we consider the prospects for the development of potential preventative treatments of AD in the DS population and their evaluation.

## Background

**Fig. 1:**
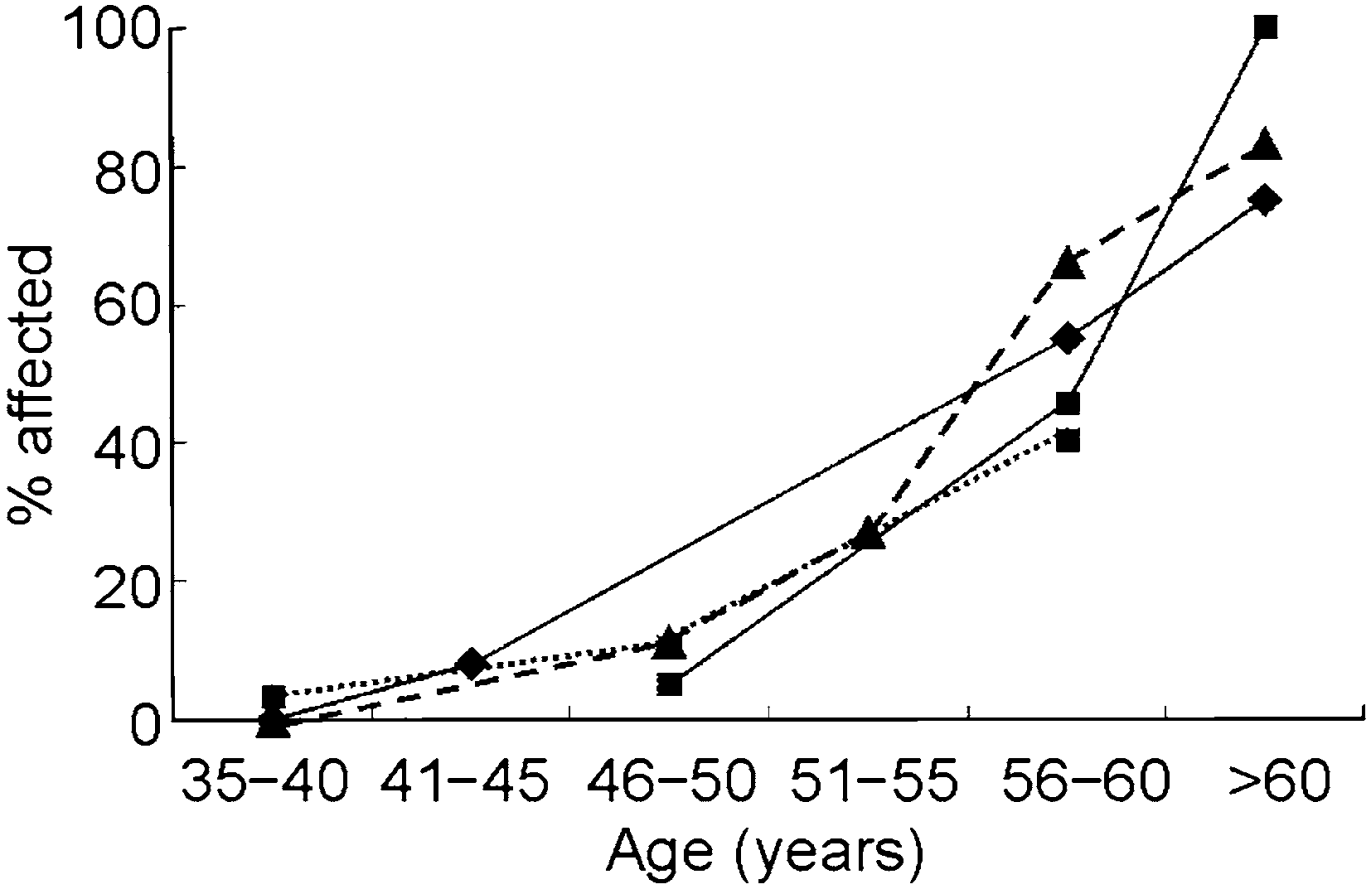
Age-specific prevalence of dementia in adults with Down’s syndrome [9]. *Line with filled diamond* data from Lai and Williams [6]; *dot with triangle* data from Visser et al. [7]; *line with filled square* data from Lai et al. [11]; *line with filled dot* data from Holland et al. [8]

Just over 20 years after the first description by Alzheimer of the characteristic neuropathology of extracellular plaques and intracellular neurofibrillary tangles associated with ‘senile dementia’, the first report of a similar neuropathology in people with DS was published [1]. Similar findings from further neuropathological studies were subsequently reported and later in the 20th century a number of cross-sectional clinical studies found that with increasing age many but not all people with DS were at risk for cognitive and functional decline not obviously dissimilar to that observed in the typically developing population with Alzheimer’s disease (AD) [2]. Over this period, the mean life expectancy for people with DS continued to improve [3] reaching an average of 60 years [4]. Consequently there has been an increase in the prevalence of age-related morbidities such as dementia. The overall prevalence of dementia in adults with DS is estimated at [1] 6.8 % with an increase with age from 8.9 % in individuals up to 49 years to 32.1 % in individuals from 55 to 59 years old [5]. However, the exact age-related percentages differ between studies with some reporting prevalence rates of 50 % or more in people with DS in their 50 s [6–8] (see Fig. 1) with other genetic and possible environmental factors influencing risk [9–11].

More recent clinical studies have focussed on the early characteristics and course of dementia in people with DS [12, 13] and, given that the diagnosis of dementia may be difficult in people with pre-existing intellectual disabilities, efforts have also been made to improve the reliability of diagnosis [14, 15]. When these various studies are considered together it is clear that there is a discrepancy, with most neuropathological studies reporting that almost 100 % of people with DS over the age of 35 have the neuropathology of AD, whereas in contrast clinical studies have found that not everyone with DS develops the clinical symptoms of dementia with increasing age [16, 17]. Furthermore, unlike the general population, memory loss is not invariably the earliest reported clinical feature. Prior to clinically diagnosed dementia or shortly after the clinical features are apparent, myoclonic or generalised seizures [18] may develop together with the onset of behavioural changes, and deterioration in performance on executive function tasks and working memory. Later in the course of dementia, deterioration in language use and comprehension and loss of functional abilities are characteristic of the subsequent course of dementia in people with DS. The mean age of onset of diagnosed AD in people with DS is reported at around 54 years [19].

Building on these earlier clinical studies advances in neuroimaging are now part of a new wave of research, which has focussed on understanding the mechanisms that underpin the relationship between a neurodevelopmental disorder (DS), on the one hand, and an illness with its onset in later life (AD), on the other. The aim of this review is to consider the challenges and possibilities of developing treatments to prevent AD in people with DS.

## Methodology

A thorough search of the Cochrane Library was performed to ensure that there were no other previous reviews published on the exact same topic. The initial literature search was performed on the PUBMED database using the keywords Alzheimer’s disease OR dementia AND biomarkers AND Down syndrome with limiters of full-text, English, written over the last 10 years and journal articles. Sixty-two articles were found and 31 were selected due to relevance to the topic. The same keywords were used in a MEDLINE database search in a different order (Down syndrome AND Alzheimer’s disease OR dementia AND biomarkers), which provided a result of 68 articles. Only five (excluding duplicates) were related to this research topic, and these were selected. Then, a different search was performed in PUBMED using the keywords Alzheimer’s disease AND treatment AND Down syndrome with the limiters of journal articles, full text, written in the last 10 years and English. One hundred and eighty-four articles were found, and 35 were selected (excluding the duplicates). Again, using the same keywords in MEDLINE and the limiters being linked full text, abstract available and English language, the search provided 12 articles, and three were selected (excluding duplicates). Based on the selected articles, a search for more specific literature was performed from their reference lists, gathering 33 articles from secondary sources. After excluding duplicates, screening the articles to meet the criteria and excluding full-text articles that did not relate to the topic of this literature review, in total, the present study compiled 39 articles related to possible preventative therapies for AD and 34 articles on the biomarkers topic (see “Appendix”).

### AD pathogenesis in DS: the amyloid cascade hypothesis

In the 1990s it was proposed that the depositions of β-amyloid (Aβ) protein and amyloid plaque formation in the brain were the main causes of the subsequent additional neuropathological hallmarks of AD, which are neurofibrillary tangles, vascular damage, an inflammatory response, and neuronal cell death [20, 21]. This theory is still the basis for much research and treatment developments in AD [22]. The association between early onset AD and mutations in the amyloid precursor protein (APP) and presenilin genes [23] has been central to the promotion of the amyloid cascade hypothesis. The observations in DS and the high risk of early onset AD have also provided important support for this hypothesis, given that the gene for APP is located on chromosome 21 [24] and therefore along with other genes on chromosome 21 it is inherited in triplicate by people with DS [25]. Much is now known about the amyloid pathway with the subsequent cleavage of APP by two enzymes β and γ-secretase creating the soluble product of Aβ [26]. Other factors, both in the general population and in people with DS, contribute to the extent of Aβ pathology, such as the status of the apolipoprotein E (ApoE) genotype. When ApoE4 (ε4) is present in DS, the risk for AD is even higher, with a two-fold increase in the amyloid load deposited in the brain [27, 28]. In contrast, when ε2 allele is present, it is associated with increased longevity and the absence of dementia [29]. Neuro-inflammation is also known to play a role in the pathogenesis of AD, especially through microglia activation [30], and it may be more influential in DS as genes regulating inflammatory processes, such as *mir*-155 and the s100 calcium-binding protein beta (S100B), are located on chromosome 21 [25]. These latter gene products are thought to impact cognitive impairment and neurodegeneration [31], as it has been proposed that inflammatory processes accelerate the development of AD in DS [32].

### The development of treatments

Pharmacological approaches to the treatment of AD in people with DS have been based largely on the cholinergic hypothesis of AD and the use of anticholinesterase inhibitors. In DS these early pharmacological treatments were found to ameliorate to a limited extent the symptoms of AD, but did not affect its subsequent course [33]. In the general population, in addition to pharmacological approaches, attention has also focussed on aspects of life style and modification of specific risk factors that may alter the disease course. These have included: exercise [34], blood pressure [35], obesity [36], diabetes [37], and the use of statins [38]. These factors have not been assessed in people with DS although, interestingly, there has been one proof of concept trial of statins in people with DS that showed a potential positive effect on age-related cognitive decline [39].

Treatments targeting the amyloid pathway aim to stop the development and progression of the disease, and hence prevent it from developing [40–43]. Rafii [44] has argued that disease-modifying treatments must therefore target prodromal and pre-clinical stages of AD and be initiated before the full symptoms of dementia appear and significant levels of neurodegeneration become established. Barage and Sonawane [45] reviewed the theories on the pathogenesis of AD and the current studies on factors that may interfere in the process. Inhibitors of cleaving enzymes, such as BACE and γ-secretase, have been proposed as potential modifiers of the amyloid cascade and, consequently, the β-amyloid deposition in the brain. Below, we briefly review four approaches that target pathways that may influence the extent of amyloid deposition and then consider the challenge of any treatment trial.

#### BACE inhibitors

β-Secretases (BACEs) cleave APP at its β-site, producing Aß after an additional cleavage by γ-secretase. Mutations in APP that occur close to this β-site are known to be triggers for the production of Aβ and some toxic species of Aβ, including Aβ42, leading to an early onset dementia [46]. BACE1 has been found to be up-regulated in trisomy 21 cells, resulting in an increased production of Aβ [47]. Inhibiting BACE1 could, therefore, be a potential therapeutic target for AD in DS. The gene for BACE2, a homologue of BACE1, which is located on chromosome 21, however, has no relationship in humans to APP cleavage [48], although one in vitro study with BACE2 raised the possibility that this enzyme may have a protective effect [49]. Studies using animal models have supported the idea that β-secretase inhibitors reduce the amount of amyloid protein in the brain, cerebrospinal fluid (CSF), and plasma [50, 51]. A recent study using 2-aminooxazoline and 3-azaxanthenes as BACE inhibitors in a rat model reported a significant reduction in amyloid levels in the brain and CSF [52]. However, according to Varghese [26] there are still no inhibitors that have been found to lower Aβ in the brain or CSF in humans.

#### γ-Secretase modulators

The γ-Secretase enzyme is the catalyst for the last stage of cleavage of β-amyloid precursor protein leading to the release of the Aβ peptide [53]. In 1999 De Strooper and Konig initiated a Phase I clinical trial of a γ-secretase inhibitor [54]. The trial ended because of toxicity but since then there have continued to be attempts to develop suitable modulators for this enzyme [55–58]. With some success Netzer et al. [51] used a γ-secretase (DAPT) inhibitor in a DS mouse model to lower amyloid levels and correct learning deficits. A protein that interacts with this enzyme and is part of the amyloidogenic pathway, the γ-secretase activating protein (GSAP), has also been studied in people with DS, and has been found to facilitate β-amyloid production without having a toxic effect, so its inhibition could be a target for development [59].

#### Anti-inflammatory drugs

Because the deposits of amyloid protein and the neurofibrillary tangles activate an inflammatory response in the brain [60], which leads to an exacerbation of the pathogenesis of AD [61], several clinical trials using non-steroidal anti-inflammatory drugs (NSAIDs) are currently being undertaken [61–63]. This group of anti-inflammatory agents has been shown to prevent human Aβ aggregation in vitro [64]. However, a study that looked at the effects of the drugs Rofecoxib and Naproxen in participants with mild to moderate AD reported that they did not reduce cognitive decline [65]. Using mouse models of DS, Cuello et al. [66] reported that the anti-inflammatory agent, minocycline, improved behavioural problems and reduced early inflammatory factors and Aβ protein in the brain, suggesting that this approach could be a therapeutic strategy.

#### Immunotherapy

Immunotherapy is also under investigation using different approaches, such as vaccines against synthetic Aβ42, fragments of Aβ protein conjugated to proteins, and also passive immunisation with antibodies [67]. In 1999 Schenk et al. succeeded in preventing the formation of Aβ plaques by immunising young transgenic AD mice that over-expressed APP, suggesting the possibility that this immunisation could also be used in humans to prevent AD [68]. The clinical trial that followed this discovery had to be stopped when 6 % of its participants developed a sub-acute meningoencephalitis related to the Aβ42 immunisation [69]. However, subsequent analysis of the trial showed that the 30 participants who had developed Aβ42 antibodies progressed at a slower rate [70]. Currently, there are three peptides in phase 2 clinical trials [71–73]. In the context of DS, these vaccines have been used in mouse models and have been shown to improve cognitive deficits, and also to prevent cholinergic neuronal atrophy without obvious side effects [74]. In terms of passive immunotherapy, there are four antibodies—solanezumab, gantenerumab, crenezumab, and aducanumab—that are currently in phase 2 or 3 clinical trials for AD investigating effects on outcomes including amyloid binding and on different biomarkers [75–81].

Thus, for people with DS in which excess Aβ production leads in part to AD, therapies aimed at the amyloid pathways to reduce production, increase elimination, or modifying its effects are available. However, to date, no such trials have been done in patients with DS.

### Biomarkers as proxy outcome measures of AD in DS

**Fig. 2:**
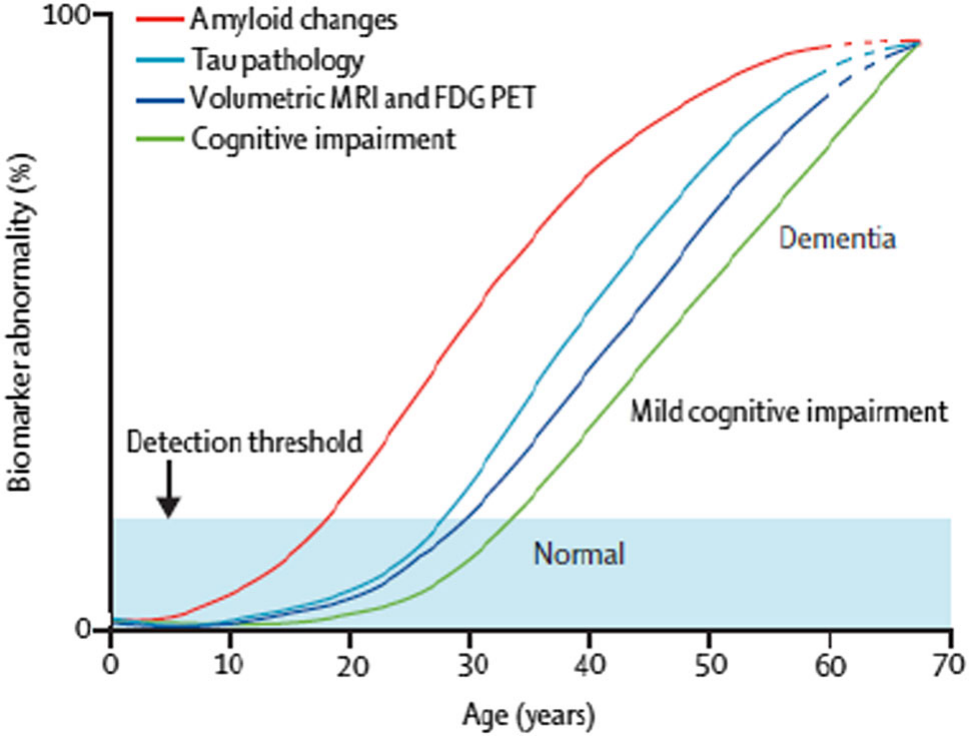
Hypothetical model for development of dementia in people with Down’s syndrome [81]

Figure 2 summarises the proposed time course of neuropathological and clinical changes across age in people with DS (based on the model by Jack and Holtzman [82]). There is a 20-year or longer period of time between the first neuropathological abnormalities and the development of AD symptoms, which means that measures other than clinical ones are needed for the trial of preventive therapies.

The Down Syndrome Biomarker Initiative (DSBI) reported by Rafii et al. [83] has investigated different types of biomarkers, including: retinal amyloid imaging, PET neuroimaging, and blood markers, and their relationship to neuropsychological findings in people with DS. We consider some of these below.

#### Plasma biomarkers

Increased levels of plasma Aβ42, thought to be the toxic species of APP, has been associated with mutations in the APP and presenilin genes, which themselves are associated with early onset of AD [84] and have been found to be correlated with cognitive decline [85]. In 2001 Schupf et al. reported that there was a sixfold increase of Aβ in the plasma of adults with DS, compared to age-matched people without DS. Both species of the β-amyloid protein (Aβ42 and Aβ40) appeared to be significantly higher in the plasma of individuals with DS, and levels may relate to the stage of the progression of the disease [86]. A decline in levels of Aβ42 and in the Aβ42/Aβ40 ratio with an increase in Aβ40 in people with DS could be indicators of AD [87]. Recently, Coppus et al. [88] published another study on plasma β-amyloid biomarkers, and observed a strong relationship between high plasma concentration of Aβ42 and Aβ40 and the risk of dementia in individuals with DS, suggesting the possibility of using them as predictors for dementia.

#### Cerebrospinal fluid (CSF) biomarkers

Different kinds of CSF biomarkers have also been studied for more than a decade in individuals with AD, the most frequent being biomarkers for amyloid and tau pathology [89–91]. In a recent study in the general population, it was observed that levels of Aβ42 in CSF decrease at least 5–10 years before someone with Mild Cognitive Impairment (MCI) converts to AD, while other molecules like T-tau and P-tau appear to be later markers of the disease [92]. Fortea et al. [93] studied CSF biomarkers in people with DS with dementia compared to those without. The preliminary results of this study showed that most people with DS over 30 years of age had at least one altered biomarker, normally Aβ in CSF, independent of their clinical diagnosis. CSF Aβ levels are high in children with DS but decrease in later life, and in a study of 12 people with DS with a mean age of 41 years, levels of Aβ1-42 were reduced compared to aged matched controls, as were other endogenous peptides. In contrast, Aβ1-28 levels were increased in those with DS [94].

#### Neuroimaging investigations (PET ligand-based, MRI, and FDG-PET neuroimaging)

Neuroimaging techniques, such as magnetic resonance imaging (MRI) and positron emission tomography (PET), are clearly important tools for identifying the abnormalities that AD causes in the brain. In 2005, Teipel and Hampel, using high-resolution MRI to measure atrophy in different regions of the brain of participants with DS, reported that age-related cerebral atrophy in people with DS happened before the onset of clinical signs of the disease [95]. A different MRI study looking at DS participants, with and without AD, found evidence that individuals with AD had significantly smaller medial temporal and striatal regions than those without AD, and thus could represent potential cerebral markers of dementia [96]. The same researchers a year later (2010), using MRI neuroimaging, reported accelerated atrophy in some regions of the DS brain, which was associated with an increased risk of dementia [97].

Different tracer compounds for the in vivo imaging of brain amyloid, neurofibrillary tangles, and activated microglia have been developed for use in PET scans [91]. One group recently evidenced a significant relationship between age and higher levels of amyloid deposition (PiB retention) in neocortical regions in asymptomatic participants with DS [98]. However, they also found no significant differences between positive PiB binding on such a scan and lower scores in neuropsychological tests, suggesting that DS adults have a tolerance for the deposition of Aβ. Amyloid binding was also first observed in the striatum in people with DS, a region also commonly affected in sporadic early onset AD [99]. In the case of DS this was observed around the age of 40, and was associated with dementia and cognitive decline [100].

Another study with a group of DS participants at risk of dementia was undertaken using FDG-PET [101]. This study showed a correlation between a higher glucose rate in middle-aged DS adults without AD and a lower rate in some regions of the brain when dementia is present. Research using Florbetapir-PET for amyloid binding, together with other imaging techniques, found an association between DS individuals with dementia and an increased fibrillar β-amyloid deposition, a lower cerebral metabolic rate for glucose, and reduced grey matter volume, in comparison with healthy controls and people with DS without dementia. This method could potentially be used to track neuropathological changes in pre-clinical stages of AD in adults with DS [102], but these changes do occur relatively late with respect to the onset of neuropathology.

#### Electroencephalography (EEG)

Compared to other possible biomarkers, neurophysiological measures, such as EEG, have received less attention, especially in the DS population. A meta-analysis that summarised the diagnostic accuracy of EEG diagnosis in dementia concluded that the EEG was not a sufficiently reliable tool for use in clinical practice for the diagnosis of MCI [103]. However, Jackson and Snyder [104] analysed the applicability of quantitative EEG as a marker of prodromal impairment and the progression of the disease, reporting that such changes were a reliable and sensitive biomarker of MCI and AD. In respect to research in DS, in 1993 one study used quantitative EEG to investigate the relationship between such measures and neuropsychological performance. They compared findings between the DS participants and people with probable AD [105], concluding that studies with people with DS of different ages could enable the development of a model of the progression of AD. Another article reported that in people with DS a reduction in the dominant occipital rhythm was related to AD and that the frequency of the same region’s activity decreased at the onset of cognitive deterioration [106]. In 2015 Salem et al., using quantitative EEG, noticed a significant decrease in the centroid frequency in people with DS and AD compared to those without any cognitive decline [107]. The conclusion of the study suggested that EEG might serve as a potential diagnostic tool, for enabling the prediction of AD, although they also emphasised the need for more studies.

#### Detectable changes in the eye

As the optic nerve and retinal nerve fibres have similar embryological origins as the central nervous system, studies have been published relating AD to abnormalities in these structures. Using a linear discriminant function (LDF) for multiple retinal measurements taken using a Fourier domain optical coherence tomography (OCT), it was found that the LDF was a better predictor of AD than any single measurement [108]. Other studies have reported a reduction in the thickness of the parapapillary and macular retinal nerve fiber layer (RNFL) and in macular volume, measured using OCT, in patients with AD and also a relationship between the loss of macular volume and the severity of AD [109]. Using this same instrument, different studies have also reported similar reductions in the RNFL thickness [110, 111] at different stages of the disease. Such a relatively non-invasive technique might therefore contribute towards an early diagnosis of AD [112, 113]. A study in people with DS using similar techniques is in progress. Investigation of the lenses in individuals with DS revealed the accumulation of Aβ aggregates, characterising the beginning of a cataract forming process [114] similar to that which has been seen in the lenses of people with AD without DS [115]. The pilot study from the Down Syndrome Biomarker Initiative (DSBI) observed amyloid plaques on retinal imaging, using a Neurovision retina high density scan, but found no correlations between retinal amyloid and cognitive deficits [83].

## Discussion

People with DS are at high risk of developing Alzheimer’s dementia relatively early in life. The most likely explanation for this risk is the ‘amyloid cascade hypothesis’. Thus, people with DS are an ideal group for investigating AD and also likely to benefit from new treatments that are developed for this condition. However, most of the medications available at the moment are symptomatic. The BACE inhibitors, 2-aminooxazoline and 3-azaxanthene, and γ-secretase modulators, DAPT, have been proven to reduce Aβ in mouse models but are still being trialled in humans with AD. Active Aβ42 immunotherapy tested in mouse models of DS has shown improvements in cognitive function and reduction in neuronal atrophy, without obvious side effects. At the same time, different methods of passive immunotherapy are currently in Phase 2/3 clinical trials for AD. Anti-inflammatory drugs (NSAIDs) were recently reported in a published meta-analysis as being ineffective in AD, even though their use in mice models of DS proved to be beneficial in reducing cerebral Aβ protein deposition.

The identification of early biomarkers for AD in people with DS is important as such proxy markers for the disease process will be necessary if preventative, as opposed to symptomatic, treatments are ultimately to be evaluated over an acceptable time scale. However, despite advances in this area evidence is needed that existing biomarkers have sufficient sensitivity and specificity to predict the development of AD in DS. Plasma and CSF markers (Aβ species), along with eye changes, still require further research. MRI and PET approaches have shown some promise, but the disease process is already advanced when such changes are easily seen using these techniques. Finally, studies using EEG have suggested that it might be a potential tool for the early detection and monitoring of the pre-symptomatic and subsequent course of AD in DS.

Since the pathological changes of AD are present before the onset of symptoms in people with DS, research on treatments for this disease need to focus on triggers and steps in the amyloid pathway, reducing the production or enhancing the clearance of Aβ protein in the brain and, consequently, arresting or delaying the development of the disease, assuming that it is correct that it is excess amyloid and the subsequent cascade of neuropathological events that are driving this process. Also it is crucial to identify biomarkers for AD in this population so as to be able to determine the efficacy of any new treatments early in the course of the underlying disease process and well before the AD-related pathology and cerebral atrophy have become established.

## Appendix

Appendix: PRISMA flowchart of methodology [116].

## References

[1] Struwe F (1929). Histopathologische Untersuchungen über entstehung und wesen der senilen Plaques. Zeitschrift für die gesamte Neurologie und Psychiatrie.

[2] Oliver C, Holland AJ (1986). Down’s syndrome and Alzheimer’s disease: a review. Psychol Med.

[3] Bittles AH, Glasson EJ (2004). Clinical, social, and ethical implications of changing life expectancy in Down syndrome. Dev Med Child Neurol.

[4] Torr J, Strydom A, Patti P, Jokinen N (2010). Aging in Down syndrome: morbidity and mortality. J Policy Pract Intellect Disabil.

[5] Coppus A, Evenhuis H, Verberne GJ, Visser F, Van Gool P, Eikelenboom P, Van Duijin C (2006). Dementia and mortality in persons with Down’s syndrome. J Intell Disabil Res.

[6] Lai F, Williams S (1989). A prospective study of Alzheimer disease in Down syndrome. Arch Neurol.

[7] Visser FE, Aldenkamp AP, van Huffelen AC, Kuilman M (1997). Prospective study of the prevalence of Alzheimer-type dementia in institutionalized individuals with Down syndrome. Am J Ment Retard.

[8] Holland AJ, Hon J, Huppert FA, Stevens F, Watson P (1998). Population-based study of the prevalence and presentation of dementia in adults with Down’s syndrome. Br J Psychiatry.

[9] Schupf N, Sergievsky GH (2002). Genetic and host factors for dementia in Down’s syndrome. Br J Psychiatry.

[10] Sekijima Y, Ikeda S, Tokuda T, Satoh S, Hidaka H, Hidaka E, Ishikawa M, Yanagisawa N (1998). Prevalence of dementia of Alzheimer type and apolipoprotein E phenotypes in aged patients with Down’s syndrome. Eur Neurol.

[11] Lai F, Kamman E, Rebeck GW, Anderson A, Chen Y, Nixon RA (1999). APOE genotype and gender effects on Alzheimer disease in 100 adults with Down syndrome. Neurology.

[12] Ball SL, Holland AJ, Huppert FA, Treppner P, Watson P, Hon J (2006). Personality and behaviour changes mark the early stages of Alzheimer’s disease in adults with Down’s syndrome: findings from a prospective population-based study. Int J Geriatr Psychiatry.

[13] Ball SL, Holland AJ, Treppner P, Watson PC, Huppert FA (2008). Executive dysfunction and its association with personality and behaviour changes in the development of Alzheimer’s disease in adults with Down syndrome and mild to moderate learning disabilities. Br J Clin Psychol.

[14] Nieuwenhuis-Mark RE (2009). Diagnosing Alzheimer’s dementia in Down syndrome: problems and possible solutions. Res Dev Disabil.

[15] Ball SL, Holland AJ, Huppert FA, Treppner P, Watson P, Hon J (2004). The modified CAMDEX informant interview is a valid and reliable tool for use in the diagnosis of dementia in adults with Down’s syndrome. J Intell Disabil Res.

[16] Wisniewski KE, Wisniewski HM, Wen GY (1985). Occurrence of neuropathological changes and dementia of Alzheimer’s disease in Down’s syndrome. Ann Neurol.

[17] Mann DMA, Royston MC, Ravindra CR (1990). Some morphometric observations on the brains of patients with Down’s syndrome: their relationship to age and dementia. J Neurol Sci.

[18] Tyrrell J, Cosgrave M, McCarron M, McPherson J, Calvert J, Kelly A, McLaughlin M, Gill M, Lawlor BA (2001). Dementia in people with Down’s syndrome. Int J Geriat Psychiat.

[19] Crayton L, Oliver C, Holland A, Bradbury J, Hall S (1998). The neuropsychological assessment of age related cognitive deficits in adults with Down syndrome. J Appl Res Intellect Disab.

[20] Hardy JA, Higgins GA (1992). Alzheimer’s disease—the amyloid cascade hypothesis. Science.

[21] Selkoe DJ (1994). Alzheimer’s disease: a central role for amyloid. J Neuropathol Exp Neurol.

[22] Korczyn AD (2008). The amyloid cascade hypothesis. Alzheimer’s Dement.

[23] Hardy J (1997). Amyloid, the presenilins and Alzheimer’s disease. Trends Neurosci.

[24] Goldgaber D, Lerman MI, Mcbride WO, Saffiotti U, Gajdusek DC (1987). Isolation, characterization, and chromosomal localization of human brain cDNA clones coding for the precursor of the amyloid of brain in Alzheimer’s disease, Down’s syndrome and aging. J Neural Transm S.

[25] Wiseman FK, Al-Janabi T, Hardy J, Karmiloff-Smith A, Nizetic D, Tybulewics VLJ, Fisher EMC, Strydom A (2015). A genetic cause of Alzheimer disease: mechanistic insights from Down syndrome. Nat Rev Neurosci.

[26] Varghese J (2010). BACE lead target for orchestrated therapy of Alzheimer’s disease.

[27] Hyman BT (1995). Neuropathological changes in Down’s syndrome hippocampal formation. Arch Neurol.

[28] Patel A, Rees SD, Kelly MA, Bain SC, Barnett AH, Thalitaya D, Prasher VP (2011). Association of variants within APOE, SORL1, RUNX1, BACE1 and ALDH18A1 with dementia in Alzheimer’s disease in subjects with Down syndrome. Neurosci Lett.

[29] Royston M, Mann D, Pickering-Brown S, Owen F, Perry R, Raghavan R, Khin-IMu C, Tyrer S, Day K, Crook R, Hardy J, Roberts GW (1994). Apolipoprotein E ε2 allele promotes longevity and protects patients with Down’s syndrome from dementia. Neuroreport.

[30] Xiang Z, Haroutunian V, Ho L, Purohit D, Pasinetti GM (2006). Microglia activation in the brain as inflammatory biomarker of Alzheimer’s disease neuropathology and clinical dementia. Dis Mark.

[31] Wilcock DM (2012). Neuroinflammation in the aging Down syndrome brain; lessons from Alzheimer’s disease. Curr Gerontol Geriatr Res.

[32] Lott IT, Head E (2005). Alzheimer disease and Down syndrome: factors in pathogenesis. Neurobiol Aging.

[33] Prasher VP, Huxley A, Haque MS (2002). A 24-week, double-blind, placebo-controlled trial of donepezil in patients with Down syndrome and Alzheimer’s disease—pilot study. Int J Geriatr Psychiatry.

[34] Law LLF, Barnett F, Yau MK, Gray MA (2014). Effects of functional tasks exercise on older adults with cognitive impairment at risk of Alzheimer’s disease: a randomised controlled trial. Age Ageing.

[35] Rouch L, Cestac P, Hanon O, Cool C, Helmer C, Bouhanick B, Chamontin B, Dartiques JF, Vellas B, Andrieu S (2015). Antihypertensive drugs, prevention of cognitive decline and dementia: a systematic review of observational studies, randomized controlled trials and meta-analyses, with discussion of potential mechanisms. CNS Drugs.

[36] Kilian AJ, Arnoldussen IAC, Gustafson DR (2014). Adipokines: a link between obesity and dementia?. Lancet Neurol.

[37] Fox C, Kilvert A (2014). Diabetes and dementia. Pract Diabetes.

[38] McGuinness B, Craig D, Bullock R, Passmore P (2009). Statins for the prevention of dementia. Cochrane Database Syst Rev.

[39] Cooper SA, Caslake M, Evans J, Hassiotis A, Jahoda A, McConnachie A, Morrison J, Ring H, Starr J, Stiles C, Sullivan F (2014). Toward onset prevention of cognitive decline in adults with Down syndrome (the TOP-COG study): study protocol for a randomized controlled trial. Trials.

[40] Hardy J (2009). The amyloid hypothesis for Alzheimer’s disease: a critical reappraisal. J Neurochem.

[41] Reitz C (2012). Alzheimer’s disease and the amyloid cascade hypothesis: a critical review. Int J Alzheimer’s Dis.

[42] Armstrong RA (2014). A critical analysis of the ‘amyloid cascade hypothesis’. Folia Neuropathol.

[43] Morris GP, Clark IA, Vissel B (2014). Inconsistencies and controversies surrounding the amyloid hypothesis of Alzheimer’s disease. Acta Neuropathol.

[44] Rafii MS (2014). Pro: are we ready to translate Alzheimer’s disease modifying therapies to people with Down syndrome?. Alzheimer’s Res Ther.

[45] Barage SH, Sonawane KD (2015). Amyloid cascade hypothesis: pathogenesis and therapeutic strategies in Alzheimer’s disease. Neuropeptides.

[46] Selkoe D (2001). Alzheimer’s disease: genes, proteins, and therapy. Physiol Rev.

[47] Sun X, Tong Y, Qing H, Chen CH, Song W (2006). Increased BACE1 maturation contributes to the pathogenesis of Alzheimer’s disease in Down syndrome. FASEB J.

[48] Ballard C, Mobley W, Hardy J, Williams G, Corbett A (2016). Dementia in Down syndrome. Lancet Neurol.

[49] Sun X, He G, Song W (2006). BACE2, as a novel APP theta-secretase, is not responsible for the pathogenesis of Alzheimer’s disease in Down syndrome. FASEB J.

[50] Ghosh AK, Brindisi M, Tang J (2012). Developing β-secretase inhibitors for treatment of Alzheimer’s disease. J Neurochem.

[51] Netzer WJ, Powell C, Nong Y, Blundell J, Wong L, Duff K, Flajolet M, Greengard P (2010). Lowering β-amyloid levels rescues learning and memory in a Down syndrome model. PLoS One.

[52] Chen JJ, Qingyian L, Chester Y (2015). Development of 2-aminooxazoline 3-azaxanthenes as orally efficacious β-secretase inhibitors for the potential treatment of Alzheimer’s disease. Bioorg Med Chem Lett.

[53] Golde TE (2005). The Aβ hypothesis: leading us to rationally-designed therapeutic strategies for the treatment or prevention of Alzheimer disease. Brain Pathol.

[54] De Strooper B, Konig G (1999). Alzheimer’s disease: a firm base for drug development. Nature.

[55] He G, Luo W, Li P (2010). Gamma-secretase activating protein is a therapeutic target for Alzheimer’s disease. Nature.

[56] Li H, Qin J, Dhondi P (2013). The discovery of fused oxadiazepines as gamma secretase modulators for treatment of Alzheimer’s disease. Bioorg Med Chem Lett.

[57] Velter AI, Bischoff FP, Berthelot D (2014). Anilinotriazoles as potent gamma secretase modulators. Bioorg Med Chem Lett.

[58] Chu J, Li JG, Joshi YB, Giannopoulos PF, Hoffman NE, Madesh M, Praticò D (2015). Gamma secretase-activating protein is a substrate for caspase-3: implications for Alzheimer’s disease. Biol Psychiatry.

[59] Chu J, Wisniewski T, Praticò D (2016). GATA1-mediated transcriptional regulation of the γ-secretase activating protein increases Aβ formation in Down syndrome. Ann Neurol.

[60] Wenk GL (2006). Neuropathologic changes in Alzheimer’s disease: potential targets for treatment. J Clin Psychiatry.

[61] Akiyama H, Barger S, Barnum S (2000). Inflammation and Alzheimer’s disease. Neurobiol Aging.

[62] Prati F, Bartolini M, Simoni E, De Simone A, Pinto A, Andrisano V, Bolognesi ML (2013). Quinones bearing non-steroidal anti-inflammatory fragments as multitarget ligands for Alzheimer’s disease. Bioorg Med Chem Lett.

[63] Xu W (2015). Meta-analysis of modifiable risk factors for Alzheimer’s disease. J Neurol Neurosurg Psychiatry.

[64] Thomas T, Nadackal GT, Thomas K (2001). Aspirin and non-steroidal anti-inflammatory drugs inhibit amyloid-β aggregation. NeuroReport.

[65] Aisen PS, Schafer KA, Grundman M, Pfeiffer E, Sano M, Davis KL, Farlow MR, Jin S, Thomas RG, Thal LJ (2003). Effects of rofecoxib or naproxen vs placebo on Alzheimer disease progression. J Am Med Assoc.

[66] Cuello AC, Ferretti MT, Leon WC, Iulita MF, Melis T, Ducatenzeiler A, Bruno MA, Canneva F (2010). Early-stage inflammation and experimental therapy in transgenic models of the alzheimer-like amyloid pathology. Neurodegener Dis.

[67] Delrieu J, Ousset PJ, Caillaud C, Vellas B (2012). ‘Clinical trials in Alzheimer’s disease’: immunotherapy approaches. J Neurochem.

[68] Schenk D, Barbour R, Dunn W (1999). Immunization with amyloid-β attenuates Alzheimer disease-like pathology in the PDAPP mouse. Nature.

[69] Orgogozo JM, Gilman S, Dartiques JF (2003). Subacute meningoencephalitis in a subset of patients with AD after Aβ42 immunization. Neurology.

[70] Hock C, Konietzko U, Streffer JR (2003). Antibodies against [beta]-amyloid slow cognitive decline in Alzheimer’s disease. Neuron.

[71] Lambracht-Washington D, Rosenberg RN (2013). Advances in the development of vaccines for Alzheimer’s disease. Discov Med.

[72] Schneeberger A, Mandler M, Otawa O, Zauner W, Mattner F, Schmidt W (2009). Development of Affitope vaccines for Alzheimer’s disease (AD)—from concept to clinical testing. Aging.

[73] Winblad B, Andreasen N, Minthon L (2012). Safety, tolerability, and antibody response of active Aβ immunotherapy with CAD106 in patients with Alzheimer’s disease: randomised, double-blind, placebo-controlled, first-in-human study. Lancet Neurol.

[74] Belichenko PV, Madani R, Rey-Bellet L (2016). An anti-β-amyloid vaccine for treating cognitive deficits in a mouse model of down syndrome. PLoS One.

[75] Blennow K, Zetterberg H, Rinne JO, Salloway S, Wei J, Black R, Grundman M, Liu E (2012). Effect of immunotherapy with bapineuzumab on cerebrospinal fluid biomarker levels in patients with mild to moderate Alzheimer disease. Arch Neurol.

[76] Bohrmann B, Baumann K, Benz J (2012). Gantenerumab: a novel human anti-Aβ antibody demonstrates sustained cerebral amyloid-β binding and elicits cell-mediated removal of human amyloid-β. J Alzheimers Dis.

[77] Farlow M, Arnold SE, Van Dyck CH (2012). Safety and biomarker effects of solanezumab in patients with Alzheimer’s disease. Alzheimers Dement.

[78] Shayan G, Adamiak B, Relkin NR, Lee KH (2012). Longitudinal analysis of novel Alzheimer’s disease proteomic cerebrospinal fluid biomarkers during intravenous immunoglobulin therapy. Electrophoresis.

[79] Relkin N, Bettger L, Tsakanikas D, Ravdin L (2012). Three-year follow-up on the IVIg for Alzheimer’s phase II study. Alzheimers Dement.

[80] Dodel R, Rominger A, Bartenstein P (2013). Intravenous immunoglobulin for treatment of mild-to-moderate Alzheimer’s disease: a phase 2, randomised, double-blind, placebo-controlled, dose-finding trial. Lancet Neurol.

[81] Sevigny J, Chiao P, Bussière T (2016). The antibody aducanumab reduces Aβ plaques in Alzheimer’s disease. Nature.

[82] Jack CR, Holtzman DM (2013). Biomarker modelling of Alzheimer’s disease. Neuron.

[83] Rafii MS, Wishnek H, Brewer JB, Donohue MC, Ness S, Mobley WC, Aisen PS, Rissman RA (2015). The Down syndrome biomarker initiative (DSBI) pilot: proof of concept for deep phenotyping of Alzheimer’s disease biomarkers in Down syndrome. Front Behav Neurosci.

[84] Scheuner D, Eckman C, Jensen M (1996). Secreted amyloid beta-protein similar to that in the senile plaques of Alzheimer’s disease is increased in vivo by the presenilin 1 and 2 and APP mutations linked to familial Alzheimer’s disease. Nat Med.

[85] Naslund J, Haroutunian V, Mohs R, Davis KL, Davies P, Greengard P, Buxbaum JD (2000). Correlation between elevated levels of amyloid B-peptide in the brain and cognitive decline. JAMA.

[86] Schupf N, Patel B, Silverman W, Zigman WB, Zhong N, Tycko B, Mehta D, Mayeux R (2001). Elevated plasma amyloid β-peptide 1–42 and onset of dementia in adults with Down syndrome. Neurosci Lett.

[87] Schupf N, Zigman WB, Tang MX, Pang D, Mayeux R, Mehta PD, Silverman W (2010). Change in plasma Aβ peptides and onset of dementia in adults with Down syndrome. Neurology.

[88] Coppus AMW, Schuur M, Vergeer J, Janssens AC, Oostra BA, Verbeek MM, Van Duijn CM (2012). Plasma β amyloid and the risk of Alzheimer’s disease in Down syndrome. Neurobiol Aging.

[89] Lewczuk P, Kamrowski-Kruck H, Peters O (2008). Soluble amyloid precursor proteins in the cerebrospinal fluid as novel potential biomarkers of Alzheimer’s disease: a multicentre study. Mol Psychiatry.

[90] Mattsson N, Blennow K, Zetterberg H (2009). CSF biomarkers. Ann N Y Acad Sci.

[91] Craig-Schapiro R, Fagan AM, Holtzman DM (2009). Biomarkers of Alzheimer’s disease. Neurobiol Dis.

[92] Buchhave P, Minthon L, Zetterberg H, Wallin AK, Blennow K, Hansson O (2012). Cerebrospinal fluid levels of β-amyloid 1-42, but not of tau, are fully changed already 5–10 Years before the onset of Alzheimer dementia. Arch Gen Psychiat.

[93] Fortea J, Benejam B, Alcolea D (2014). Core Alzheimer’s disease CSF biomarkers in Down syndrome. Alzheimers Dement.

[94] Portelius E, Soininen H, Andreasson U (2014). Exploring Alzheimer molecular pathology in Down’s syndrome cerebrospinal fluid. Neurodegener Dis.

[95] Teipel SJ, Hampel H (2005). Neuroanatomy of Down syndrome in vivo: a model of preclinical Alzheimer’s disease. Behav Genet.

[96] Beacher F, Daly E, Simmons A, Prasher V, Morris R, Robinson C, Lovestone S, Murphy K, Muprhy DG (2009). Alzheimer’s disease and Down’s syndrome: an in vivo MRI study. Psychol Med.

[97] Beacher F, Daly E, Simmons A, Prasher V, Morris R, Robinson C, Lovestone S, Murphy K, Muprhy DG (2010). Brain anatomy and ageing in non-demented adults with Down’s syndrome: an in vivo MRI study. Psychol Med.

[98] Hartley SL (2014). Cognitive functioning in relation to brain amyloid-β in healthy adults with Down syndrome. Brain.

[99] Klunk WE, Engler H, Nordberg A (2004). Imaging brain amyloid in Alzheimer’s disease with Pittsburgh Compound-B. Ann Neurol.

[100] Annus T, Wilson LR, Hong YT (2015). The pattern of amyloid accumulation in the brains of adults with Down syndrome. Alzheimer’s Dement.

[101] Haier RJ, Head K, Head E, Lott IT (2008). Neuroimaging of individuals with Down’s syndrome at-risk for dementia: evidence for possible compensatory events. Neuroimage.

[102] Sabbagh MN (2015). Florbetapir PET, FDG PET, and MRI in Down syndrome individuals with and without Alzheimer’s dementia. Alzheimers Dement.

[103] Jelic V, Kowalski JE (2009). Evidence-based evaluation of diagnostic accuracy of resting EEG in dementia and mild cognitive impairment. Clin EEG Neurosci.

[104] Jackson CE, Snyder PJ (2008). Electroencephalography and event-related potentials as biomarkers of mild cognitive impairment and mild Alzheimer’s disease. Alzheimer’s Dement.

[105] Soininem H, Partanen J, Jousmaki V, Helkala EL, Vanhanen M, Majuri S, Kaski M, Hartikainen P, Riekkinen P (1993). Age-related cognitive decline and electroencephalogram slowing in Down’s syndrome as a model of Alzheimer’s disease. Neuroscience.

[106] Menendez M (2005). Down syndrome, Alzheimer’s disease and seizures. Brain Dev jpn.

[107] Salem LC, Sabers A, Kjaer TW (2015). Quantitative electroencephalography as a diagnostic tool for Alzheimer’s dementia in adults with down syndrome. Dement Geriatr Cogn.

[108] Larrosa JM, Garcia-Martin E, Bamboo MP, Pinilla J, Polo V, Otin S, Satue M, Herrero R, Pablo LE (2014). Potential new diagnostic tool for Alzheimer’s disease using a linear discriminant function for fourier domain optical coherence tomography. Invest Ophthalmol Vis Sci.

[109] Iseri PK, Altinas O, Tokay T, Yuksel N (2006). Relationship between cognitive impairment and retinal morphological and visual functional abnormalities in Alzheimer disease. J Neuro Ophthalmol.

[110] Berisha F (2007). Retinal abnormalities in early Alzheimer’s disease. Invest Ophthalmol Vis Sci.

[111] Kromer R (2014). Detection of retinal nerve fiber layer defects in Alzheimer’s disease using SD-OCT. Front Psychiatry.

[112] Paquet C (2007). Abnormal retinal thickness in patients with mild cognitive impairment and Alzheimer’s disease. Neurosci Lett.

[113] Kesler A (2011). Retinal thickness in patients with mild cognitive impairment and Alzheimer’s disease. Clin Neurol Neurosurg.

[114] Moncaster JA, Pineda R, Moir RD (2010). Alzheimer’s disease amyloid-β links lens and brain pathology in Down syndrome. PLoS One.

[115] Goldstein LE, Muffat JA, Cherny RA (2003). Cytosolic beta-amyloid deposition and supranuclear cataracts in lenses from people with Alzheimer’s disease. Lancet.

[116] Moher D, Liberati A, Tetzlaff J, Altman DG, The PRISMA Group (2009). Preferred Reporting items for systematic reviews and meta-analyses: the PRISMA statement. PLoS Med.

